# The SENSS (Stress, Exercise, Nutrition, Sleep, Self-management) study: study protocol for a randomized controlled trial to evaluate an integrated, personalized and stepped care lifestyle approach for people with Parkinson’s disease

**DOI:** 10.3389/fneur.2026.1752073

**Published:** 2026-03-03

**Authors:** Ties J. Gaveel, Elbrich M. Postma, Bastiaan R. Bloem, Sirwan K. L. Darweesh, Marten Munneke, Karin Overbeek, Rick Helmich, Florian Zeevat, Meine Zijlstra, Cornelis Boersma, Sebastiaan Overeem, Angelique Pijpers, Caroline Jakimowicz, Merel M. van Gilst, Anne E. M. Speckens, Regina Thé, Gert Manhoudt, Annemiek van der Wel, Annelien A. Duits, Nienke M. de Vries

**Affiliations:** 1 Radboud University Medical Center, Department of Neurology, Center of Expertise for Parkinson and Movement Disorders, Donders Institute for Brain, Cognition and Behaviour, Nijmegen, Netherlands; 2 Health-Ecore, Zeist, Netherlands; 3 Department of Health Sciences, University Medical Center Groningen, University of Groningen, Groningen, Netherlands; 4 Department of Management Sciences, Open University, Heerlen, Netherlands; 5 Sleep Medicine Center Kempenhaeghe, Heeze, Netherlands; 6 Biomedical Diagnostics Lab, Department of Electrical Engineering, Eindhoven University of Technology, Eindhoven, Netherlands; 7 Radboud University Medical Center, Department of Psychiatry, Center of Expertise for Mindfulness, Donders Institute for Brain, Cognition and Behaviour, Nijmegen, Netherlands; 8 Development and Implementation of Decision Aids, ZorgKeuzeLab, Delft, Netherlands; 9 Patient Research Council, Dutch Parkinson Association, Utrecht, Netherlands; 10 Department of Psychiatry and Neuropsychology, Faculty of Health, Medicine and Life Sciences, School for Mental Health and Neuroscience, Maastricht University, Maastricht, Netherlands; 11 Department of Medical Psychology, Maastricht University Medical Center, Maastricht, Netherlands; 12 Department of Medical Psychology, Radboud University Medical Center, Nijmegen, Netherlands; 13 Department of Human Movement Science, University Medical Center Groningen, University of Groningen, Groningen, Netherlands

**Keywords:** exercise, lifestyle, nutrition, Parkinson’s disease, personalized, self-management, sleep, stress

## Abstract

**Background:**

Lifestyle interventions have potential to support people with Parkinson’s disease (PD) in self-managing their disease and improving quality of life. Growing evidence suggests positive effects of singular lifestyle interventions, such as physical activity, stress or nutrition. However, several challenges remain. First, despite potential additive and perhaps even synergistic effects, research on combining lifestyle interventions is limited. Second, lifestyle interventions are not routinely addressed as part of standard medical care. Finally, there are significant challenges related to changing behavior and adherence to lifestyle interventions. This study aims to evaluate the effectiveness and cost-effectiveness of an integrated, personalized and stepped care lifestyle approach for people with PD.

**Methods:**

We will perform a 12-month single-blind randomized controlled trial. We aim to include 256 people with PD, randomized in a 1:1 ratio into a control and intervention group. Both groups receive a clinical assessment with a lifestyle coach and can partake in usual care. The intervention group receives an additional lifestyle intervention, consisting of guidance and interventions on different lifestyle domains: stress, exercise, nutrition, sleep and self-management (the SENSS approach). This intervention is offered remotely according to a stepped care model to personalize the intervention to the participants’ needs and abilities. The primary endpoint is the between-group difference in quality of life at 12 months (Parkinson’s Disease Questionnaire-39). Secondary endpoints include between-group differences in PD symptoms, health-related outcomes, self-management and personal goals. Cost-effectiveness and the experiences of participants and healthcare professionals will also be explored.

**Discussion:**

This study evaluates the effectiveness of an integrated personalized lifestyle intervention for people with PD on both clinical and socio-economic outcomes. We expect this intervention to improve quality of life and self-management of people with PD, without increasing healthcare costs. We also expect to offer valuable insights into how such an intervention can be integrated into current daily care for people with PD.

**Clinical trial registration:**

ClinicalTrials.gov, identifier NCT06669455.

## 1 Introduction

### 1.1 Background and rationale

Parkinson’s disease (PD) is one of the most common neurological disorders, with a prevalence of almost 12 million people worldwide (1). People with PD often experience both motor and non-motor symptoms, which greatly impact their quality of life (QoL) (2, 3). Currently, there is no cure for PD. Medical management, including medication or deep brain surgery, can provide symptomatic relief, but this is often insufficient to alleviate all symptoms (4). In addition, the responsiveness to dopaminergic medication differs between individuals and across symptoms, and the effects wear off over time (5). Therefore, lifestyle interventions are increasingly recognized as a valuable additional way to support people with PD (6).

Lifestyle includes six pillars: physical activity, nutrition, stress management, restorative sleep, social connection and risky substance avoidance (7). Among these, physical activity, optimal nutrition, stress management and sleep have received most attention in research and clinical practice. Exercise attenuates motor- and non-motor symptoms and increases QoL, and may perhaps affect disease progression (8–10). There is also growing excitement about the potential role of other lifestyle interventions. Dietary interventions can have multiple benefits. For example, optimal fluid and fiber intake may reduce constipation and thereby increase medication effectiveness (11). In addition, a well-balanced intake of proteins and dopaminergic medication is important: proteins can compete with dopaminergic medication by causing malabsorption in the gut and brain, thus limiting its efficacy (12). At the same time, proteins are essential for body composition and muscle preservation. A lack of protein consumption may cause malnutrition and underweight (13). Underweight is a well-known indicator of negative health outcomes and also increases the risk of mortality (14). Another important factor related to nutrition is the gut microbiome, which plays a role in the pathophysiology of PD by impacting on, e.g., mitochondrial functioning, inflammation and immune responses (13). Although the evidence from robust clinical trials remains limited, there is enough reason to motivate people with PD to adhere to a healthy diet with adequate intake of macronutrients as suggested by organizations such as the World Health Organization (WHO) (15). The other lifestyle domains have been studied less well. However, emerging evidence shows that stress-reducing interventions, such as mind–body exercises and mindfulness-based interventions (MBIs), can reduce, e.g., anxiety and depression for people with PD, thus improving QoL (16–19). Moreover, positive effects of sleep hygiene education in people with PD with sleep disturbances have been found (20, 21).

So far, no studies have investigated the effects of interventions including multiple lifestyle domains in people with PD. While all domains are expected to have positive effects in isolation, they may even have a larger, additive and perhaps even synergistic effect when delivered in combination (6). Moreover, no studies have evaluated the cost-effectiveness of offering lifestyle interventions in PD.

In the absence of robust studies on effectiveness and cost-effectiveness, lifestyle interventions are currently not routinely offered as part of standard medical care. Consequently, people with PD receive hardly any guidance related to lifestyle changes. Based on interviews and our own clinical experience, we identified that many healthcare professionals in the Netherlands are not trained to provide lifestyle information or to offer personalized coaching (22, 23). Lifestyle coaches have the potential to play a role as a central coordinator of lifestyle interventions with expertise on all lifestyle domains and capable of coaching toward behavioral change (24, 25). The potential of lifestyle coaches has been shown in other chronic conditions (24, 25). Of note, changing lifestyle behavior is an enormous challenge for people with PD, especially when one needs to sustainably change multiple lifestyle aspects (26, 27). Therefore, a personalized approach, taking into account potential barriers related to lifestyle behavior is needed.

Motivated by these considerations, we here propose to combine different lifestyle domains into one approach as part of a personalized and stepped care lifestyle intervention. This approach, called the SENSS approach (stress, exercise, nutrition, sleep and self-management), is based on a combination of previous pilot initiatives (22, 28–32). A stepped care approach, with limited guidance when possible but with access to more intensive treatment when necessary, will deliver personalized care without further straining an already overburdened healthcare system. We expect that such a stepped care approach minimizes the extra costs for providing lifestyle guidance, yet lead to benefits of a healthy lifestyle. We expect this approach to be associated with a reduction of (long-term) healthcare costs, an increased productivity of people with PD and their caregivers and an improved QoL.

### 1.2 Objectives

Our primary objective is to evaluate the effect of the SENSS approach on QoL in people with PD. Our secondary objective is to evaluate the effects of SENSS on PD motor- and non-motor symptoms, self-management, overall health and attaining personal lifestyle goals. A third objective is to analyze the costs and (socio-)economic impact of SENSS compared to current usual care. Our fourth objective is to evaluate the experiences of participants, lifestyle coaches and healthcare professionals with the SENSS approach.

**Figure 1:**
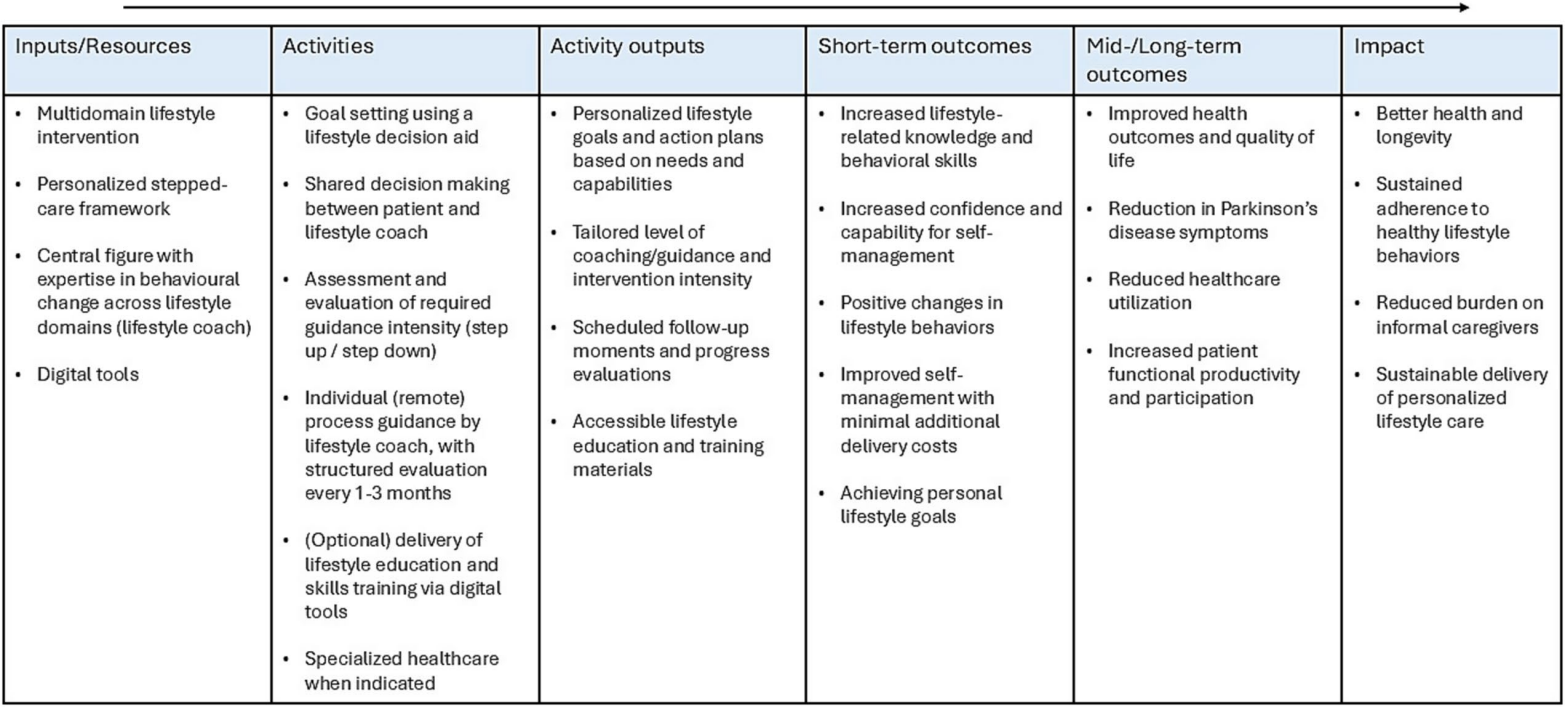
Logic model SENSS.

Figure 1 provides an overview of the key elements of the SENSS intervention and the expected outcomes.

## 2 Methods

### 2.1 Trial design

**Figure 2:**
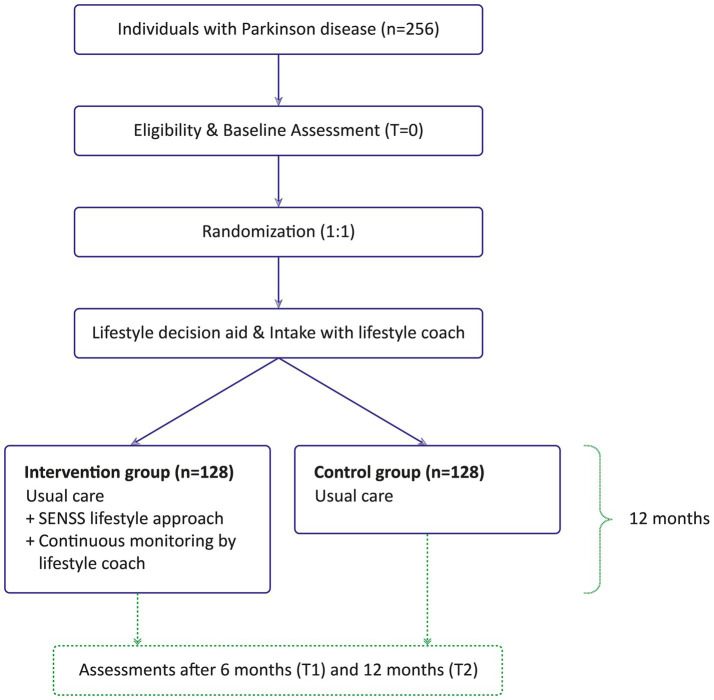
Overview of study design.

The SENSS study is a 12-month single-blind, randomized controlled trial (RCT). The intervention group receives a combined lifestyle intervention (SENSS approach) after a personalized intake and with continuous monitoring by a lifestyle coach. The control group only receives the personalized intake with a lifestyle coach, but continues with usual care. A total of 256 individuals with PD will be included, equally randomized into the control and intervention group. Participants complete three remote assessments: at baseline, 6 months, and 12 months follow-up. An overview of the study design is provided in Figure 2.

### 2.2 Study setting

The study will be performed by the Radboud University Medical Center (Radboudumc), Nijmegen, the Netherlands. The assessments and interventions of the study will be provided remotely. The study will be conducted in the Netherlands. People with PD will be recruited through a number of open recruitment strategies (see the section on recruitment strategies).

### 2.3 Eligibility criteria

Inclusion criteria are: a confirmed diagnosis of PD by a neurologist according to established diagnostic criteria, and being able to read and understand the Dutch language. The exclusion criteria are: cognitive impairments that do not allow to complete the questionnaire, as judged by the researcher during the screening call; not in possession of or no access to an electronic device that allows for (video) calls with a lifestyle coach and to complete the questionnaires; planned surgery or an expected major change in treatment plan; participation in other intervention trials during the participation of the SENSS study.

### 2.4 Who will take informed consent?

If a potential participant shows interest in the study, an assessor from the research team sends an information letter and informed consent through mail. After 2 weeks the assessor will contact the participant to answer questions, to check for eligibility and to discuss consent. When the participant is eligible and willing to participate, written informed consent will be obtained by signing the consent form and sending it to the research team by post. If an informal caregiver is available who is willing to fill out questionnaires, (s)he will receive an information letter and sign an informed consent as well. Participation of the informal caregiver is not mandatory for the participant to be included and does not influence the intervention in any way.

### 2.5 Intervention

The intervention is a personalized and stepped care lifestyle intervention (SENSS) that combines five different and complementary lifestyle domains (stress, exercise, nutrition, sleep and self-management). In this intervention, self-management complements four important lifestyle domains, as it plays an overarching role in lifestyle behavior.

In the SENSS approach, participants receive guidance by a trained lifestyle coach. The title of lifestyle coach is not legally protected in the Netherlands. To ensure intervention quality, lifestyle coaches delivering the intervention are required to be affiliated with the Dutch professional association for lifestyle coaches (Beroepsvereniging Leefstijlcoaches Nederland; BLCN). BLCN affiliation requires compliance to strict admission standards and ongoing professional development. This includes holding at least a bachelor’s degree at a university of applied sciences level and having completed a BLCN-accredited postgraduate lifestyle coaching program, or having successfully completed an individual accreditation pathway (33). For this trial, two certified and dedicated lifestyle coaches will provide the intervention. Participants are divided pragmatically between the two lifestyle coaches, based on coach availability at the time of enrolment.

**Figure 3:**
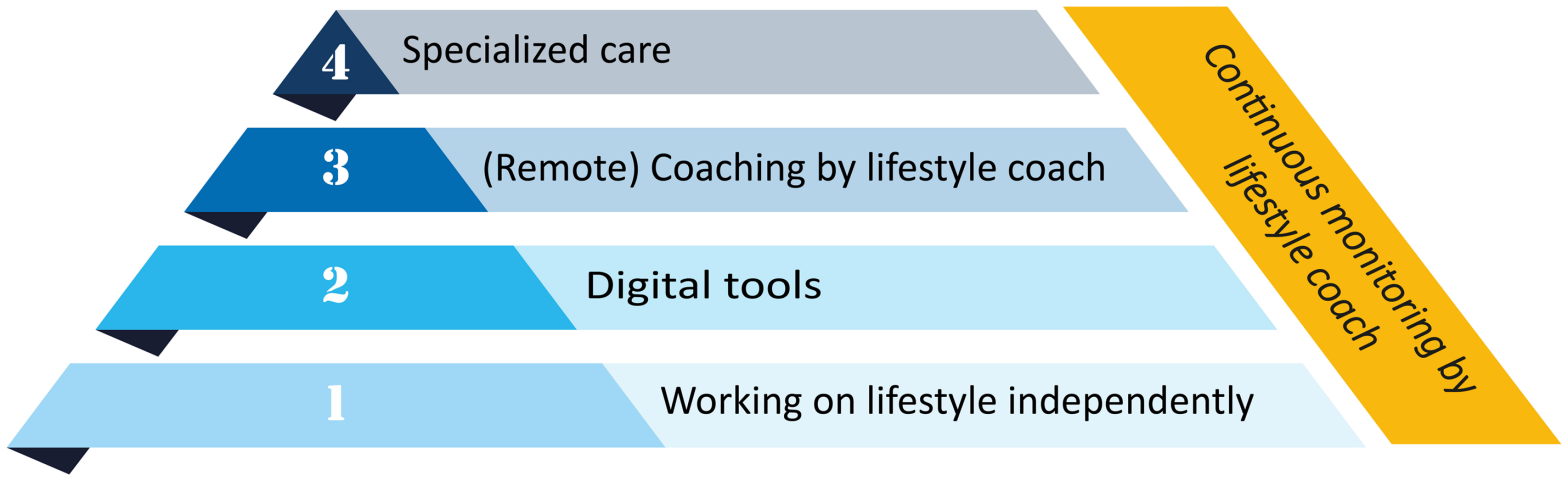
The stepped care model.

The SENSS approach starts with goal setting using a lifestyle decision aid, previously developed by our group in accordance with the International Patient Decision Aid Standards (22, 34). The Parkinson lifestyle decision aid (35) is a web-based tool. The tool includes information and questions that support people with PD to identify their needs in relation to lifestyle, set goals, make a plan, and determine the type of support they may need. The questions range from mapping the current situation in relation to their lifestyle, to clarifying their values, preferences and goals for all five lifestyle domains and identifying the needs for support to reach sustainable changes in lifestyle. The decision aid concludes with a summary of the answers given. This summary serves as a starting point for the consultation with the lifestyle coach, during which shared decision making is used to determine which lifestyle domain(s) to prioritize and what level of support is appropriate. For the participants in the intervention group, this level of support is further specified in a stepped care model (Figure 3).

The stepped care model is used to provide just the right amount of guidance. By following the steps in the model, people with PD are encouraged to work on their lifestyle goals independently, supported by technological tools when possible (self-management). Professional support is provided when needed, based on participant reported needs and the lifestyle coach’s professional judgment. The approach is highly personalized, meaning participants do not have to follow a fixed sequence of steps. They can move between steps in either direction throughout the intervention. Importantly, a lifestyle coach will continuously monitor adherence and progress, and discusses with the participant whether changes are needed in either the lifestyle domain they are working on or in the level of support. The frequency of monitoring is decided through shared decision making by the lifestyle coach and participant, within a range of once every month to once every 3 months. In the following paragraphs the different steps will be described in more detail.

#### 2.5.1 Step 1: working on lifestyle independently

Participants in step 1 are willing and able to work on their personal lifestyle goals independently, or with support from the people in their immediate environment. The lifestyle coach monitors the progress on the agreed intervals, as described in the previous paragraph.

#### 2.5.2 Step 2: digital tools

Step 2 consists of support by digital tools. Tools have been selected for each lifestyle domain, based on previous work, mostly by members of our consortium. The lifestyle coach is responsible for monitoring the progress and actual usage of the tools in the domains stress, exercise, nutrition and self-management. For the online sleep training, a dedicated sleep therapist (a psychologist trained in behavioral sleep medicine) is involved. This approach is taken because sleep is only minimally addressed in current curricula for lifestyle coaches, and when it is included, specialized knowledge on PD-related sleep disorders is often lacking.

The following tools are available for this study: MindDistrict (mindfulness based stress reduction) (36, 37), STEPWISE (exercise) (38), Nutritional app by the Dutch “Voedingscentrum” (nutrition) (39), i-Sleep (sleep) (32), Parkinson in Balance (self-management) (30), and an online Parkinson Yoga library (multiple domains, including exercise and stress). More detailed information about the individual tools can be found in Supplementary material 1.

#### 2.5.3 Step 3: remote coaching by lifestyle coach

When the participant needs more guidance than the online tools can provide, the next step includes remote personal coaching by the lifestyle coach (coaching mode). This differs from the continuous guidance provided throughout the stepped care model (guidance mode) in the frequency and content of the sessions. In the guidance mode, the lifestyle coach monitors the progress throughout and evaluates whether other approaches are needed. The progress is evaluated by discussing interim goals with participants. These evaluations are documented by the coach for clinical purposes and not included as research data. In the coaching mode, active coaching is provided to support the participant in changing behavior within a specific lifestyle domain. The number of sessions will be decided upon in mutual agreement with the participant, but this will be more frequent than in the guidance mode. Although the focus shifts to one-on-one guidance, the digital tools from step 2 can still be used to support the lifestyle change. Lifestyle coaching is, at this point, not part of the formal care pathways for people with PD. Lifestyle coaches in this study are trained to reach a sufficient level of PD-specific expertise, analogous to the way we train other allied health professionals as part of the Dutch national ParkinsonNet approach (40, 41). Examples of PD specific issues that may interfere with adherence to lifestyle advice include frontal executive problems, difficulties with dual tasking, fatigue and complex changes in the effectiveness of symptomatic pharmacotherapy. An essential part of the training of lifestyle coaches is to clarify which other professionals within ParkinsonNet can offer further detailed guidance, if the lifestyle coach feels insufficiently equipped to address a particular issue himself or herself (see step 4). Furthermore, they are trained to recognize PD related red flags (such as unintended weight loss or balance problems) requiring referral to healthcare professionals (step 4).

#### 2.5.4 Step 4: treatment by specialized healthcare professional

Some participants may experience specific barriers or problems, such as PD related red flags, that hinder making lifestyle changes and require more expertise. These participants can be referred to specialized healthcare professionals (e.g., a physiotherapist, dietician, psychologist or social worker). In the Netherlands, specialized allied healthcare is organized as part of the Dutch ParkinsonNet approach, with national coverage. Cost-effectiveness of ParkinsonNet has been shown in multiple studies (42, 43). Because ParkinsonNet currently does not include somnological treatment, a sleep medicine center (Kempenhaeghe) is involved, where specialized care for sleep disorders can be provided, and in which extensive experience is available on sleep and PD. In this study, all referrals to specialized care will be made through standard healthcare procedures in the Netherlands (i.e., the general practitioner overseeing the participant’s care).

#### 2.5.5 Strategies to improve adherence to intervention

Several strategies to improve adherence are an inherent part of the SENSS approach, starting with the Parkinson lifestyle decision aid. The intervention is fully personalized to the participants’ needs and abilities, and can be adapted to changes along the way. Additionally, adherence to the intervention is monitored by the lifestyle coach during the structural contact moments.

#### 2.5.6 Relevant concomitant care permitted or prohibited during the trial

Both the control and intervention group will receive usual care. Participants in the control group are allowed to use any intervention or digital tool that is (publicly) available that may support them in a healthy lifestyle. However, most digital tools that are used in the intervention group are not publicly available, and therefore not accessible by the control group (MindDistrict, STEPWISE, i-Sleep, Parkinson in Balance, and the online Parkinson Yoga library are not publicly available). Apart from those digital tools, participants are not restricted in any type of care they use.

### 2.6 Outcomes

#### 2.6.1 Primary outcome

The primary outcome is the between-group difference in QoL from baseline to 12 months (end of intervention) measured with the Parkinson’s Disease Questionnaire-39 (PDQ-39) (44). The PDQ-39 is the most widely used QoL scale in PD, frequently used as the outcome measure, e.g., in trials on deep brain stimulation, multidisciplinary care and allied health interventions (45, 46). This questionnaire assesses the impact of PD on a person’s QoL. It consists of 39 questions across eight dimensions of daily living including relationships, social situations and communication. Item scores are filled out on a 5-point scale from “never” to “always.” Both the total score of the PDQ-39 and the scores for each of the eight dimensions are considered primary outcomes.

#### 2.6.2 Secondary outcomes

To address the second study objective, one or more questionnaires were included per lifestyle domain when a single instrument did not capture all relevant constructs. Furthermore, the secondary outcome measures focus on personal goals, general health and PD symptoms. The secondary outcomes of this study are presented in Table 1, including the constructs and used outcome measures. All questionnaires are patient reported outcomes, administered at baseline (T0), after 6 months (T1) and after 12 months (T2). Participants will receive the following questionnaires: health-related quality of life (EuroQol 5D-5L) (47–49), non-motor and motor aspects of PD in daily living (MDS-Unified Parkinson’s Disease Rating Scale-IB and -II) (50, 51), fatigue (Fatigue Severity Scale) (52), evaluation of lifestyle goals (Visual Analogue Scale) (53), diet quality (Dutch Healthy Diet Index 2015) (54), symptoms of anxiety and depression (Hospital Anxiety and Depression Scale) (55), physical activity (LASA Physical Activity Questionnaire) (56), insomnia (Insomnia Severity Index) (57), daytime sleepiness (Epsworth Sleepiness Scale) (58–60), general perceived self-efficacy (General Self-Efficacy Scale) (61, 62), disease and impairment acceptance (Acceptance of Disease and Impairments Questionnaire) (63), coping (Ways of Coping Questionnaire) (64, 65), and self-management (Patient Activation Measure) (66). Additionally, a wrist monitor (Empatica EmbracePlus) (67) will be worn 1 week at baseline (T0) and 1 week after 12 months (T2) to collect data on physical activity and sleep quality. Due to a limited number of available monitors, approximately two-thirds of the participants will wear the wrist monitor. The EmbracePlus uses electrodermal activity, photoplethysmogram (PPG), digital skin temperature and accelerometer sensors to collect data. Raw sensor data is accessible through an online dashboard. The EmbracePlus will be worn on the arm that is least affected by PD symptoms, to optimize monitoring of physical activity and minimize interference from symptoms such as tremor and bradykinesia.

**Table 1:** Overview primary and secondary outcome measures.

(Lifestyle) domain	Construct	Outcome measure	T0	T1	T2
General	Quality of life (primary outcome)	PDQ-39	**⨯**	**⨯**	**⨯**
	Health related quality of life	EQ-5D-5L	**⨯**	**⨯**	**⨯**
	Parkinson’s disease symptoms	MDS-UPDRS 1b & 2	**⨯**	**⨯**	**⨯**
	Fatigue	FSS	**⨯**	**⨯**	**⨯**
	Goal attainment	VAS	**⨯**	—	**⨯**
Nutrition	Diet quality	DHD15-index	**⨯**	**⨯**	**⨯**
Stress	Mood (anxiety and depression)	HADS	**⨯**	**⨯**	**⨯**
Exercise	Physical activity level	LAPAQ	**⨯**	**⨯**	**⨯**
	Physical activity	Wearable sensor	**⨯**	—	**⨯**
Sleep	Insomnia	ISI	**⨯**	**⨯**	**⨯**
	Sleepiness	ESS	**⨯**	**⨯**	**⨯**
	Sleep quality	Wearable sensor	**⨯**	—	**⨯**
Self-management	Self-efficacy	GSES	**⨯**	**⨯**	**⨯**
	Illness acceptance	ADIQ	**⨯**	**⨯**	**⨯**
	Coping	WCQ	**⨯**	—	—
	Self-management	PAM	**⨯**	**⨯**	**⨯**

T0 = Baseline measurement, T1 = 6 month measurement, T2 = 12 month measurement (end of intervention), PDQ-39, Parkinson’s Disease Questionnaire-39; EQ-5D-5L, EuroQol 5D-5L; MDS-UPDRS, MDS-Unified Parkinson’s Disease Rating Scale; FSS, Fatigue Severity Scale; VAS, Visual Analogue Scale; DHD15-index, Dutch Healthy Diet Index 2015; HADS, Hospital Anxiety and Depression Scale; LAPAQ, LASA Physical Activity Questionnaire; ISI, Insomnia Severity Index; ESS, Epsworth Sleepiness Scale; GSES, General Self-Efficacy Scale; ADIQ, Acceptance of Disease and Impairments Questionnaire; WCQ, Ways of Coping Questionnaire; PAM, Patient Activation Measure.

##### 2.6.2.1 Cost-effectiveness

We will perform a cost-effectiveness analysis from a societal perspective. Data on the frequency and duration of healthcare service utilization will be collected through a questionnaire at T1 and T2. The healthcare services include, but are not limited to, the use of allied healthcare, hospital, social care services (e.g., physiotherapists, neurologist, social workers). The questionnaire allows participants to report additional healthcare disciplines that are not listed. In addition, the use of assistive devices purchased during the study period will be recorded, including whether these were reimbursed through insurance. Additionally, to expand the cost-effectiveness analysis beyond healthcare utilization, participants receive questions regarding their work and productivity status at all time points (T0, T1, T2). In this context, productivity refers to both labor force participation and on-the-job performance (68, 69). For each participant in the intervention group, the lifestyle coach will record the amount of guidance provided, the use of digital tools, and the extent of lifestyle coaching. These elements will be included in the economic evaluation. Specialized care (step 4) is evaluated using a questionnaire on healthcare utilization at T1 and T2. The QoL related outcomes resulting from the EQ-5D-5L questionnaire enables the derivation of utility scores and the estimation of quality-adjusted life years (QALYs), which will be incorporated into the cost-effectiveness analysis.

##### 2.6.2.2 Informal caregiver questionnaires

To be able to provide more detailed information for the (socio)economic analysis, we also include informal caregivers. They will be asked to fill out several questionnaires at T0, T1 and T2. The questionnaires that will be sent online include the Caregiver Strain Index (CSI) (70), the EQ-5D-5L questionnaire, and several questions about their working life (e.g., productivity, and informal care leave).

##### 2.6.2.3 Process evaluation

To support future implementation, we will perform a process evaluation alongside the RCT. In this process evaluation we will evaluate the experiences of participants, lifestyle coaches and healthcare professionals. We will use questionnaires and interviews for this process analysis. For a process evaluation on the use of the lifestyle decision aid, various log data are collected (i.e., number of sessions, session duration).

### 2.7 Participant timeline

Initially, an assessor from the research team sends an information letter and informed consent to potential participants. Two weeks later, a phone call is planned with the participants, during which they have the opportunity to ask questions. If they are still willing to participate, participants undergo a brief cognitive assessment over the phone to determine eligibility and informed consent will be obtained. Upon signing the informed consent, participants will receive multiple questionnaires for the baseline assessment which they can complete at home, at their own pace within two weeks. Reminder emails will be sent after two and four weeks, followed by telephone contact after six weeks if questionnaires remain incomplete. After the baseline questionnaires have been returned, participants will be randomly allocated into the control or intervention group. Within two weeks after completing the questionnaires participants will receive a package. Some participants will receive a package containing a wrist monitor. The monitor will be worn one week at the start of the study and one week at the end. The package also includes a “part 2.” After the week in which the participant has worn the monitor, part 2 of the package can be opened. It contains the randomization result and login details for the lifestyle decision aid. Participants can contact a helpdesk during office hours for additional information or guidance on how to use the wrist monitor or decision aid if needed. Participants who receive a package without wrist monitor can open the randomization results and login details for the lifestyle decision aid immediately.

The lifestyle coach will schedule an intake meeting with participants two weeks after the package delivery. The participants are required to complete the lifestyle decision aid before the meeting. Participants in the intervention group schedule a follow-up meeting with the lifestyle coach during the intake, typically one to three months later, based on personal goals and needs. The control group receives no further coaching after the intake.

All participants will receive a set of questionnaires at T1 and T2. Importantly, participants will not be required to visit the research location at Radboudumc. All research procedures can be performed from their own home.

### 2.8 Sample size

Based on a recent systematic review on self-management interventions in people with PD (71) we identified two RCT’s (72, 73) that have studied a self-management intervention including lifestyle advice for people with PD using the PDQ-39 as an outcome. While the present intervention is much more comprehensive and personalized and includes more coaching and guidance when needed, we decided to conservatively use the results of those studies for our sample size calculation. Both studies found a mean between group difference of 4.15 on the PDQ-39, with a mean standard deviation of 10.55. With a significance level of 0.05 and 80%, we need 102 participants per group. Assuming 20% dropouts, we will include 128 participants per group (256 in total).

### 2.9 Recruitment

We will apply a series of previously successful recruitment strategies. First, we will send an invitation to a panel of people with PD interested in research, who are subscribed to a mailing list of our research group. They have either shown interest in research by subscribing to the mailing list or by participating in one of our previous research projects. Second, we will exploit other options to reach potential participants, including advertising and blogging on social media (LinkedIn, Facebook, X, Instagram) and the online platforms of the Parkinson Vereniging (Dutch association for people with PD), giving presentations at local and regional patient support groups, recruiting patients at our outpatient clinic at the Center of Expertise for Parkinson & Movement Disorders at the Radboudumc, and using the regional PD professional network (i.e., the Dutch ParkinsonNet with national coverage). Furthermore, word-of-mouth recruitment is encouraged via current participants and community contacts. We aim to recruit a representative study population by using multiple complementary recruitment strategies, balancing targeted approaches to reach individuals with greater lifestyle-related needs with broader recruitment efforts to ensure sufficient statistical power within the limited timeframe of the trial (74).

### 2.10 Randomization

#### 2.10.1 Sequence generation

After completing the baseline questionnaires, eligible participants will be randomly allocated to the intervention group or the control group in a 1:1 ratio. Randomization will be performed in the data management system CastorEDC (75) using random block sizes (block sizes: 4, 6, 8). We will stratify the randomization based on disease duration, categorizing participants into three groups: <5 years, 5–10 years, and >10 years since disease onset. The randomization will be performed by a researcher who is not involved in the measurements and data analysis. The study is single-blinded. The researcher performing the analyses is not aware of treatment allocation.

#### 2.10.2 Implementation and concealment mechanism

Participants will be enrolled by research assessors. The participants will be assigned by EP (project leader), who is not involved in the data collection or analysis. The participant receives a sealed envelope after completing the baseline questionnaires. This envelope can be opened after finishing the baseline assessment of the wrist monitor.

### 2.11 Blinding

The researcher performing the analyses is fully blinded from allocation. In addition, we aim to partially blind the participants by informing them that they are either in a guided lifestyle group or an independent lifestyle group, instead of naming them the intervention and control group. At the end of the study, participants will be asked to indicate which group they believe they were allocated to, in order to assess the impact. We do not foresee any reasons to unblind the participants for the coordinating researcher (TG).

### 2.12 Data collection

#### 2.12.1 Plans for assessment and collection of outcomes

The primary and most secondary outcomes in this study are collected through questionnaires completed by participants at home via the CastorEDC system. These questionnaires are sent to participants’ email addresses by the study’s researchers or assessors immediately after obtaining informed consent, and again after 6 and 12 months. Wrist sensor data will be collected by participants in their own environment. To promote data quality, the wrist sensor is accompanied by a step-by-step instruction. Additionally, the Empatica app provides real-time feedback to the participant to confirm whether the device is being worn correctly.

#### 2.12.2 Plans to promote participant retention and complete follow-up

To support participant retention, updates are sent via email newsletters to all participants twice a year. The updates will be kept general to minimize deblinding. Furthermore, to promote compliance, participants in the control group receive the publicly available lifestyle decision aid, and an intake with the lifestyle coach as a starting point. They have access to the information in the decision aid throughout the intervention. Additionally, all participants are invited to the yearly symposium for people with PD organized by our center.

### 2.13 Data management

Questionnaire data in this study is securely stored using CastorEDC, a certified data management system with a built-in audit trail. Data from the wrist monitor will be stored and made accessible through Empatica’s Carelab cloud environment, which is also certified and complies with relevant data protection (67). Within the Empatica environment, pseudonyms will be used for all participants, and no directly identifiable data will be collected or stored. Log data generated using the lifestyle decision aid is stored within the secure environment of Zorgkeuzelab (35), a member of the study consortium. This data is pseudonymized and Zorgkeuzelab does not have access to any personal information of the participants.

### 2.14 Confidentiality

Personal information will be stored in the participant management software Salesforce (Salesforce, San Francisco, CA, United States). Access to this system is restricted to the research team and protected by secure password authentication. The participants receive a pseudonym that will be stored in PIMS, a local system in the Radboudumc to manage pseudonyms and identifiers in a secured and central way, separate from the research data. The informed consents and diagnosis confirmation will be collected and stored on paper in a fireproof and locked cabinet at the Radboudumc. The data collected in this study will be archived for 15 years after the study.

### 2.15 Statistical methods

Descriptive statistics will be used to summarize baseline characteristics. Means, standard deviations, medians, ranges, counts, and percentages will be reported as appropriate, depending on the distribution and scale of the variable.

#### 2.15.1 Methods for primary and secondary outcomes

##### 2.15.1.1 Primary outcomes

The primary endpoint is the difference between groups in QoL (PDQ-39) over 12 months’ time. A linear mixed model (LMM) with repeated measurements will be used to evaluate the changes over time between the intervention and control group. The primary outcome and dependent variable is the total score of the PDQ-39, with additional models fitted for each of the eight domain scores. We include fixed effects for group, time (T0, T1 and T2) and the interaction between group and time. Sex, age, and disease duration at baseline will serve as covariates. Random effects will be included for participants to account for within-subject correlation and for lifestyle coaches to account for clustering by coach.

##### 2.15.1.2 Secondary outcomes

The secondary analyses will evaluate the between-group difference in secondary outcomes (Table 1) over 12 months using the same LMM structure as the primary analysis. In addition, the raw wrist monitor accelerometer and PPG data collected at baseline and at 12 months (each for a one-week period) will be processed to derive information on physical activity and sleep. We will use algorithms developed in-house at the Radboudumc (76) and in collaboration with Eindhoven University of Technology (TU/e) (77) to process the raw data into interpretable metrics such as total physical activity, sedentary behavior, sleep duration and sleep quality. After processing the data, average values across the one-week periods will be calculated per participant at each timepoint. The within-subject changes from baseline to 12 months will be compared between groups using a LMM with the same structure as the primary and other secondary analyses.

##### 2.15.1.3 Cost-effectiveness analysis

The cost-effectiveness analysis will be conducted from both a healthcare and societal perspective. This analysis will include direct healthcare costs, such as, resource use during the intervention and standard care, as well as broader societal costs, including caregiver burden and productivity losses. Quality-adjusted life years (QALYs) will be calculated based on responses to the EQ-5D-5L. The primary outcome of the analysis will be the Incremental Cost-Effectiveness Ratio (ICER). To account for uncertainty in model parameters, a Probabilistic Sensitivity Analysis (PSA) will be performed using Monte Carlo simulation. The results of the PSA will be visualized using a Cost-Effectiveness Plane, and a Cost-Effectiveness Acceptability Curve (CEAC), illustrating the probability of cost-effectiveness across a range of willingness-to-pay (WTP) thresholds. In addition, a Deterministic Sensitivity Analysis (DSA) will be conducted to assess robustness of the results to variations in key parameters. The results of the DSA will be presented using a tornado diagram. Several additional scenario analyses may also be performed to explore alternative assumptions and perspectives.

#### 2.15.2 Methods for additional analyses

Exploratory mediation analyses will be performed on the primary outcome (PDQ-39 total score) to assess the potential mediating role of psychosocial constructs, including general self-efficacy (GSES), coping style (WCQ), and self-management (PAM). Exploratory subgroup analyses will be conducted to investigate effects based on the specific lifestyle domain participants focused on during the intervention. Specifically, we will explore (1) whether there are differences in QoL between participants who targeted different lifestyle domains, (2) multiple lifestyle domains versus a single domain, and (3) whether participants show improvements in outcomes directly related to the domain they worked on. For example, whether those who focused on the sleep domain demonstrate an improvement in insomnia and sleep quality. We hypothesize that participants may show greater improvements in outcomes directly related to the lifestyle domain they actively targeted. Furthermore, we will perform exploratory subgroup analyses based on disease duration. Participants will be categorized into three groups: <5 years, 5–10 years, and >10 years since disease onset.

#### 2.15.3 Methods in analysis to handle protocol non-adherence and any statistical methods to handle missing data

Statistical analysis will be performed based on the intention-to-treat principle. Missing data will be handled using maximum likelihood estimation within the linear mixed-effects models, under the assumption that data are missing at random (MAR).

#### 2.15.4 Plans to give access to the full protocol, participant level-data and statistical code

The data gathered in this project will be made available upon reasonable request to the international research community via the Radboud Data Repository (RDR). All resulting publications will be published Open Access (gold open access). The statistical code will be published with restricted access and will be available upon reasonable request.

### 2.16 Oversight and monitoring

#### 2.16.1 Monitoring

Due to the nature of this study, it has been classified as not subject to the Dutch Medical Research Involving Human Subjects Act (WMO) by the local accredited Medical Research Ethics Committee (MREC). As such, the study is not required to follow the standard procedures involving an independent monitor. Nevertheless, as the coordinating center, the study team remains accountable to the sponsor and is required to adhere to all applicable laws and guidelines governing the quality of scientific research, as would be expected for WMO compliant studies. Yearly status updates on the study will be presented to the study sponsor, the funding body and the involved consortium of this study. This study is a low-risk study and therefore, a Data Safety Monitoring Board is not indicated.

#### 2.16.2 Adverse event reporting and harms

All adverse events (AEs) reported by the participants or observed by the lifestyle coach or study staff will be documented. The investigator will report all SAEs to the sponsor (Radboudumc) without undue delay after obtaining knowledge of the events by means of a SAE-form. An overview of all SAEs is provided in the annual summary report.

#### 2.16.3 Communicating protocol amendments to relevant parties

Significant amendments will be communicated to participants via email or phone and will be updated in the ClinicalTrials.gov registration. Minor protocol changes will be shared with participants twice a year through the study’s newsletter. Amendments that may lead to a change in the WMO status will be submitted to the local MREC.

### 2.17 Dissemination plans

All study participants will be informed about the study results through email. In addition, we will use communication channels of ParkinsonNet, The Dutch Parkinson Patient Association and ParkinsonNL, targeting people with PD, their caregivers and healthcare professionals. We will further promote the results at (patient) conferences, Parkinson support groups and via social media. All non-identifiable data collected during this study will be shared with the international research community via the RDR, and will be available upon reasonable request. The publications arising from this study will be made available Open Access.

## 3 Discussion

The SENSS study evaluates the effects of an integrated, personalized and stepped care lifestyle approach for people with PD, aiming to study the effects on QoL, overall health, PD symptoms and self-management over the course of 1 year. In addition, we explore the socio-economic impact of this approach compared to usual care. We expect this trial to show a positive effect of the SENSS approach on QoL, self-management and motor- and non-motor symptoms for people with PD. By using a largely remote and stepped care approach, the costs of this intervention can be kept relatively low. We therefore hypothesize that the SENSS approach will have a positive cost-effectiveness ratio. Moreover, this trial will give us valuable insights into how different lifestyle interventions can be efficiently integrated, scaled and offered within the current care landscape where the number of healthcare professionals are becoming increasingly scarce.

Our study has several strengths. First, we expect that considering the individual barriers and facilitators in a personalized approach can increase adherence to the multifaceted lifestyle intervention (78). The lifestyle coach will carefully monitor adherence throughout the intervention and will increase the frequency of contact and adjust the advice when necessary (79). Second, the intervention is provided fully remotely, making it accessible and easily scalable. Also, by using a largely remote and stepped care approach, we expect that the healthcare costs can be kept relatively low. Third, we collect raw accelerometer and PPG data to objectively measure outcomes in the participants’ own home environment. This allows us to apply open source algorithms specifically developed for people with PD (76), unlike commercially available devices that rely on built-in software designed for the general population. The algorithms are publicly available for sharing, allowing for independent replication of our findings. Fourth, our study benefits from a collaboration with a rich and multidisciplinary consortium. This consortium includes people with PD, healthcare professionals, experts on all domains of lifestyle interventions, health economists, and experts in the organization of care from different institutions throughout the Netherlands. This versatile collaboration improves the quality and potential relevance of our study, as it brings together diverse perspectives and expertise. Finally, we expect that many people require changes in multiple lifestyle domains to achieve the most benefits, but there has been limited research into the effectiveness of a combined lifestyle intervention for people with PD. Our research aims to contribute to addressing this gap in knowledge.

There are also a few potential challenges we anticipate. First, there is a risk of limited external validity. Individuals already interested in lifestyle interventions are more likely to enroll, while those less interested may be harder to reach. Analysis of participants in previous studies at our center indicates that the sex distribution aligns with the Dutch PD population, but older adults and non-native Dutch individuals are underrepresented (80). We aim to include people across all age categories and socio-economic backgrounds. This is particularly challenging since the approach is mainly based on eHealth, and offered in Dutch. To address this, we capitalize on our rich experience around diversity when including participants (81–83). We will implement proactive strategies such as targeted outreach to underserved populations and selective advertising. Additional measures include offering technical support and providing paper-based alternatives for the assessments when necessary. Moreover, due to the personalized nature of the intervention, it can be tailored to the individual participant’s needs and abilities. Second, there is a risk of selection bias. Since the control group will not receive any intervention after the intake at the start of the study, we anticipate an increased dropout risk in this group. We aim to prevent this by keeping them involved with the study by sending them biannual newsletters and invitations to the yearly symposium for people with PD organized by our center. Also, we decided to give these participants access to the information in the lifestyle decision aid, to promote compliance (84). Furthermore, we kept the burden of assessments low by performing them fully remote, restricted to questionnaires and a wrist monitor that will be sent to people’s homes. Third, there is a potential risk of contamination between the intervention and the control group. Blinding of the participants is difficult in an intervention study using a non-pharmacological intervention. We will try to get as close to blinding as possible by minimizing information about the intervention for the control group, and by naming the groups the guided and independent lifestyle interventions. We expect the impact of possible contamination to be minimal, because it is difficult to keep up with a behavioral change on the long term without (structural) guidance (85). At the end of the study, participants will be asked to indicate which group they believe they were allocated to, in order to assess the impact. Also, the control group only has access to the publicly available tools and therefore not to all tools used in the intervention group. Importantly, our present approach will reveal how the proposed intervention performs, over and above current clinical situation, including the ability of people with PD to access the available interventions themselves.

Our approach is based on several existing structures in the Dutch healthcare system and may therefore not be directly generalizable to other countries. For instance, in the Netherlands, referrals are made through the general practitioner and a national network for collaboration among professionals with expertise in PD (ParkinsonNet). We integrate our intervention within these existing structures to improve the cost-effectiveness (42, 43). Additionally, although lifestyle coaching is not yet part of usual care for individuals with PD, accredited training programs and a recognized professional association (BLCN) are available in the Netherlands. This may not be the case in other countries.

In conclusion, the SENSS approach combines several existing successful initiatives into a comprehensive personalized intervention that addresses multiple domains of lifestyle. If proven to be (cost-)effective, we envision the approach to offer a scalable solution for the promotion of sustainable lifestyle change for people with PD. In the long run, we expect our approach to yield a reduction in healthcare utilization, driven by improved self-management, better health, and increased productivity among people with PD and their caregivers.

## Trial status

The current protocol version is 2.2. The first participant was included on 25 November 2024. As of 20 November 2025, 219 participants have been randomized, of which 0 finished the study. We expect to randomize the last participant by December 2025.

## Glossary

ADIQ: Acceptance of Disease and Impairments Questionnaire
AEs: Adverse events
CBT-i: Cognitive behavioral therapy for insomnia
CSI: Caregiver Strain Index
DHD15-index: Dutch Healthy Diet Index 2015
EQ-5D-5L: EuroQol 5D-5L
ESS: Epsworth Sleepiness Scale
FSS: Fatigue Severity Scale
GSES: General Self-Efficacy Scale
HADS: Hospital Anxiety and Depression Scale
ISI: Insomnia Severity Index
LAPAQ: LASA Physical Activity Questionnaire
LMM: Linear mixed model
MBCT: Mindfulness-based cognitive therapy
MBIs: Mindfulness-based interventions
MDS: Movement Disorders Society
MDS-UPDRS: MDS-Unified Parkinson’s Disease Rating Scale
MREC: Medical Research Ethics Committee
PAM: Patient Activation Measure
PD: Parkinson’s disease
PDQ-39: Parkinson’s Disease Questionnaire-39
PPG: Photoplethysmography
QALYs: Quality-adjusted life years
QoL: Quality of life
Radboudumc: Radboud University Medical Center
RCT: Randomized controlled trial
RDR: Radboud Data Repository
SAEs: Serious adverse events
SENSS: Stress, Exercise, Nutrition, Sleep, Self-management
TU/e: Eindhoven University of Technology
VAS: Visual Analogue Scale
WCQ: Ways of Coping Questionnaire
WMO: Dutch Medical Research Involving Human Subjects Act
