# Supplementary material for: The SENSS (Stress, Exercise, Nutrition, Sleep, Self-management) study: study protocol for a randomized controlled trial to evaluate an integrated, personalized and stepped care lifestyle approach for people with Parkinson’s disease
Source: Front Neurol. 2026 Mar 3;17:1752073. doi: 10.3389/fneur.2026.1752073 (PMC12991984; doi:10.3389/fneur.2026.1752073)
Supplement: Supplementary file 1 [file Table_1.docx]

**Supplementary material 1 – Description of digital tools available in STEP 2**

*MindDistrict*

MindDistrict is a platform available as a website or app, focusing on digital mental health. The platform offers an online training that aims at reducing stress and improving mental health, based on Mindfulness-Based Cognitive Therapy (MBCT) (1, 2) .This online MBCT was developed in our own center (Radboudumc) and adapted to fit for PD, by providing extra information specific for the disease and adapting the videos to provide lived experiences by people with PD (3, 4). These adaptions have been piloted by user experts. The online training consists of 8 modules, each taking a week to complete. Participants can follow the online training at their own pace. Each module consists of theoretical background through text and videos. Additionally, the modules consist of daily exercises that are guided through audio fragments and accompanied by written explanations.

*STEPWISE*

The STEPWISE app has been developed as a motivational app to increase physical activity in people with PD. Participants receive feedback on their physical activity level and their progression towards each week’s goal (quantified by step count). Furthermore, participants will be motivated to reach their goal by a virtual coach through chat messages and challenges. The app has been developed in co-creation with people with PD and its feasibility was shown in a pilot study (5). The app is being used at this moment in a large RCT (n=450) to evaluate the physical activity levels (i.e. step counts) and clinical effects after using this app for 12 months (6).

*Nutrition apps*

To guide nutrition and promote a healthy diet, the Dutch ‘Voedingscentrum’ has developed several tools (7). In this project we will use three existing apps, that function as guiding tools to adhere to the general guidelines for a healthy diet as indicated by the ‘Gezondheidsraad’ (8, 9). Alongside these apps participants will receive a document containing disease specific information for nutrition and PD (e.g. the effects of protein on dopaminergic medication).

The apps used are ‘Mijn Eetmeter, ‘Kies Ik Gezond?’and ‘Slim Koken’. The ‘Mijn Eetmeter’ app allows a person to track their intake. Based on the intake, the app provides an analysis of the macro- and micronutrients present in the daily consumption and the total amount of consumed calories per day. The ‘ Kies Ik Gezond?’ app can be used to scan, search and compare food products. It provides an overview of the nutritional values, ingredients and whether it fits the general guidelines for a healthy diet as indicated by the ‘Gezondheidsraad’, and is found in the “Schijf van Vijf” (Dutch Food Pyramid) (33). The recipe app ‘Slim Koken’ offers over 2,000 recipes to facilitate healthy and sustainable eating by using ingredients from the "Schijf van Vijf" (Dutch Food Pyramid).

*I-Sleep*

The i-Sleep app is an online program, originally developed at the Vrije Universiteit Amsterdam in collaboration with the University of Amsterdam (Netherlands) by prof. A. van Straten and dr. J. Lancee, for the treatment of insomnia. It consists of elements commonly incorporated in face-to-face cognitive behavioral therapy for insomnia (CBT-i) such as psychoeducation, sleep hygiene, behavioral interventions (stimulus control and sleep restriction), relaxation techniques and cognitive therapy focused on restructuring negative sleep related thoughts. The online training consists of five modules, each taking a week to complete. During the modules participants receive theoretical background information, exercises and assignments. They are also asked to keep a daily sleep dairy, to (self) monitor progress and gain insight into their sleep patterns.

*Parkinson in Balance*

Parkinson in Balance is an online self-management program for people with PD. This established program is based on a self-management program for caregivers that was previously developed and tested (10): (Parkinson) Partner in Balance. The aim of the ‘patient’ version is to increase self-management and quality of life for people with PD. The tool consists of an intake session with a coach, an online period of 8 weeks, and an evaluation session with the coach. The coach is an experienced PD care professional, with training in self-management techniques and is familiar with the Parkinson in Balance program. The program is fully remote. The included modules are 1) ‘acceptance’ (including dealing diagnosis, guarantee identity, meaning in life), 2) ‘balance in activities’ (including combination with work, family life), 3) ‘lifestyle’ (exercise, nutrition, medication, self-monitoring), 4) ‘communication’(with environment), 5) ‘social interaction and support’ (including peer and professional support), 6) ‘insecurities and stress’ (coping with the disease and progression), 7) ‘self-understanding’ (including focus on the positive) and 8) ‘thinking about the future’ (including late phase, admission, end of life questions).

*(Online) Yoga*

In cooperation with Yoga4Parkinson, an online yoga intervention was developed. Yoga practice can attribute to both physical and mental health for people with PD (11, 12). The intervention consists of a library of guided videos tackling different themes throughout the weeks. At the start of the online yoga intervention participants required to go through instruction videos with safety instructions and contra-indications. Participants are free to choose the frequency and volume, but are advised to practice at least once a week, for at least two months. The guided video’s range from 30 minutes to one hour a day, and the frequency ranges from 1-5x a week. Furthermore, a daily 10 minute guided ‘Sun Salutation’ is provided and recommended.

**References**

1. Cillessen L, Johannsen M, Speckens AEM, Zachariae R. Mindfulness-based interventions for psychological and physical health outcomes in cancer patients and survivors: A systematic review and meta-analysis of randomized controlled trials. Psychooncology. 2019;28(12):2257-69.

2. Badaghi N, Buskbjerg C, Kwakkenbos L, Bosman S, Zachariae R, Speckens A. Positive health outcomes of mindfulness-based interventions for cancer patients and survivors: A systematic review and meta-analysis. Clin Psychol Rev. 2024;114:102505.

3. Badaghi N, van Kruijsbergen M, Prins J, Kelders S, Cillessen L, Compen F, et al. Effect of blended and unguided online delivery of mindfulness-based cognitive therapy versus care as usual on distress among cancer patients and survivors: protocol for the three-arm parallel randomized controlled buddy trial. BMC Psychol. 2023;11(1):21.

4. Badaghi N, van Kruijsbergen M, Speckens A, Vilé J, Prins J, Kelders S, et al. Group, Blended and Individual, Unguided Online Delivery of Mindfulness-Based Cognitive Therapy for People With Cancer: Feasibility Uncontrolled Trial. JMIR Form Res. 2024;8:e52338.

5. Schootemeijer S, de Vries NM, Darweesh SKL, Ascherio A, Schwarzschild MA, Macklin EA, et al. Promoting Physical Activity in People With Parkinson's Disease Through a Smartphone App: A Pilot Study. J Neurol Phys Ther. 2025;49(2):74-81.

6. Schootemeijer S, de Vries NM, Macklin EA, Roes KCB, Joosten H, Omberg L, et al. The STEPWISE study: study protocol for a smartphone-based exercise solution for people with Parkinson's Disease (randomized controlled trial). BMC Neurol. 2023;23(1):323.

7. Voedingscentrum. Apps en Tools 2024 [cited 2024 July 5]. Available from: <https://www.voedingscentrum.nl/nl/thema/apps-en-tools-voedingscentrum.aspx>.

8. Asseldonk MJMDv, Dicke HC, Beemt BJWvd, Berg DJvd, Borg St, Duin GM, et al. Richtlijn Diëtetiek bij de ziekte van Parkinson. Den Haag: Boom Lemma uitgevers; 2012.

9. Gezondheidsraad. Alle adviezen over voeding 2022 [cited 2024 July 5]. Available from: <https://www.gezondheidsraad.nl/onderwerpen/voeding/alle-adviezen-over-voeding>.

10. Duits AA, Boots LMM, Mulders AEP, Moonen AJH, Vugt ME. Covid Proof Self-Management Training for Caregivers of Patients with Parkinson's Disease. Movement disorders : official journal of the Movement Disorder Society. 2021;36(3):529-30.

11. Mailankody P, Varambally S, Thennarasu K, Pal PK. The Rationale of Yoga in Parkinson's Disease: A Critical Review. Neurology India. 2021;69(5):1165-75.

12. Suárez-Iglesias D, Santos L, Sanchez-Lastra MA, Ayán C. Systematic review and meta-analysis of randomised controlled trials on the effects of yoga in people with Parkinson’s disease. Disability and Rehabilitation. 2022;44(21):6210-29.
